# The Chameleon-Like Properties of Psychoactive Drugs: Examinations with HR LC-MS/MS Technology of Patients Presenting at the Emergency Department Following the Use of Synthetic Cannabinoids: A Case Series and Literature Review

**DOI:** 10.3390/bs8100088

**Published:** 2018-09-22

**Authors:** Ismail Altintop, Cigdem Karakukcu

**Affiliations:** 1 Department of Emergency Medicine, Health Science University Kayseri Training and Research Hospital, Kayseri 38010, Turkey; 2 Department of Biochemistry, Health Science University Kayseri Training and Research Hospital, Kayseri 38010, Turkey; ckarakukcu@hotmail.com

**Keywords:** synthetic cannabinoid, Emergency Department, intoxication, cannabinoid metabolites, laboratory

## Abstract

The chameleon can disguise itself in nature by taking on different colors and forms. As synthetic cannabinoids (SC) have clinically similar effects to those of several psychoactive agents, they are one of the most difficult intoxications to diagnose. The reasons for this are due to clinical variations throughout the world and the differences in symptoms having not been determined due to their similarity to the intoxication of several other drugs. The aim of this study was to obtain prospective data of patients presenting at the Emergency Department (ED) with suspected SC intoxication, and as a result of prospective examination of samples, to determine a new generation of SC use, SC types, clinical findings, and treatments. **Method:** A total of a 15 patients with suspected SC intoxication who presented at the ED of the Health Sciences University Kayseri Training and Research Hospital between January 2017 and January 2018 were examined. Samples taken prospectively from patients who were followed-up for a diagnosis of SC intoxication were examined with the HR LC-MS/MS method; SC were determined, and the test results of other psychoactive agents that were used concurrently were examined. **Conclusions:** Three significant findings emerged as a result of this study. Firstly, due to the different clinical forms of presentation at ED associated with SC use and the range of intoxications that cannot be diagnosed, advanced laboratory tests are required, in addition to routine tests for the determination of SC. Secondly, those diagnosed as having taken SC were also determined to have used it concurrently with substances that have a high potential for addiction, such as amphetamines and quetiapine. Thirdly, in regard to examples of cases presented in the literature, anti-psychotics, fluid hydration, and anxiolytics can be used as treatment options for those diagnosed with SC use.

## 1. Introduction

Cannabinoids are generally classified into three groups: natural cannabinoids, endogenous cannabinoids, and synthetic cannabinoids (SC) [1,2]. Natural cannabis is obtained from the Indian cannabis plant (Cannabis Sativa). Presently, natural cannabis is used as an antiemesis in cancer patients, a spasmolytic for patients with multiple sclerosis, an appetite enhancer for patients with AIDS, an anti inflammatory for patients with rheumatoid arthritis, and an anti-inflammatory for patients with Crohn’s disease. The best-known example of natural cannabinoids, delta 9-tetrahydrocannabinol (Δ9-THC), is the major active component in marijuana [1,2,3]. Endogenous cannabinoids are 2-arachidonylglycerol and anandamide. SC are made up of molecules created in the laboratory to mimic THC effects [2,3].

New psychoactive substances synthesized to mimic the effects of illegal drugs have become a global major healthcare concern [1,2]. SC, which is synthesized to mimic new-generation psychoactive effects, is a group exhibiting structural variations [3,4]. Pathophysiologically, the cannabinoid receptors complex is a part of the endocannabinoid system, and, to date, its two cannabinoid receptors have been identified as CB1 and CB2 [5]. CB1 receptors are responsible for the psychoactive effects of cannabinoids such as increasing emotional state, anxiety, and panic reactions [5]. CB2 receptors are seen predominantly in the marginal zone of the spleen, in the tonsils, and in immune cells [5].

During scientific studies in the 1940s that investigated the effects of cannabis and cannabinoid receptors, the first plant drug (cannabis) molecules were synthesized [6,7,8]. With easy access to various chemical components, the Internet has allowed chemists in several countries to synthesize these components. JWH-type SC were developed by the chemist John W Huffman at Clemson University in the USA in the 1990s and are a synthetic cannabinoid family known by his initials. There are known to be approximately 450 varieties of SC in the JWH group, and the most widely used on the market are JWH-18 and JWH-73 metabolites [4,6,7,8].

Following SC synthesis and identification, it was marketed under the names of “spice” and “K” and was registered in Europe in 2004 and in the USA in 2008. Generally, SC is sprayed over the plant mixture and sold as a plant mixture [9]. Despite warnings on the packaging of “not for human consumption”, it became known throughout the world in 2008 because of its psychotic effects [9,10,11].

The unwanted effects of SC consumption are nausea and vomiting, hypertension, tachycardia, seizures, cognitive changes, behavioral changes, hallucinations, acute kidney damage, and death [10]. The effects show variations when used together with side-products due to the heterogeneity of chemical analogues and simultaneous synthesis [4]. In addition to acute symptoms, dependency and withdrawal reactions associated with SC have been reported [12]. Patients have been reported to experience nightmares, hallucinations, tremor, headache, insomnia, paranoia, panic attacks, and tachycardia during withdrawal [12,13].

With an increase in the use of SC since 2014, and an increase in cases of new-generation SC substances, there has been an increase in research and studies in all clinics, but primarily in EDs. Due to the range of SC and ever-increasing different metabolites, there are significant difficulties in diagnosis and treatment [9]. These synthetic components cannot be determined in routine urine drug tests, and this creates difficulties treating patients in the ED [9]. In this case series, by reporting the clinical and laboratory data of cases presenting at our ED with new-generation SC intoxication, it was our aim to contribute information to the current literature that can be used by healthcare personnel to compare similar cases in the future. 

## 2. Materials and Methods

This case series included patients who presented at the ED of Kayseri Training and Research Hospital between January 2017 and January 2018 with a diagnosis of SC intoxication. Before starting the study, permission was granted by the Local Ethics Committee, and informed consent was obtained from the patients. On presentation of the patients, a record was made of demographic data, clinical status, hemodynamic parameters, laboratory tests, and clinical course.

In the laboratory tests, evaluation was made of the full blood count, blood glucose level, liver function tests (AST, ALT), blood urea nitrogen (BUN), creatinine (Cre), serum electrolytes (sodium) [Na+], potassium [K+], chloride [Cl], and calcium [Ca+++]). In the follow-up in the ED, the time to regaining full consciousness (h) was recorded, and treatment results were evaluated. For patients with suspected synthetic substance use, analyses were made with HR LC-MS/MS in Orbitrap Technology with respect to multiple substance use and differential diagnosis.

Informed consent was obtained from all patients from whom urine samples were taken in the ED. Within 4 min of taking the sample, the temperature of the urine was measured, samples that were not in the range of 32 to 37 °C were rejected, and a further sample was taken. The urine samples were sent to the laboratory for substance analysis, and as a result of the screening analysis, samples determined positive for toxic content were apportioned to a double tube and stored for 1 year at −20 °C. Following derivation, HR LC-MS/MS in Orbitrap Technology was applied. For confirmation analysis, the Thermo Scientific Q Exactive Mass spectrometer (Thermo Fisher Scientific, Bremen, Germany) was used combined with HR LC-MS/MS in Orbitrap Technology system (Thermo Scientific Ultimate 3000 RSLC). The samples were analyzed following the mobile phase with dilution without any need for any precipitation or online extraction process.

## 3. Results

An evaluation was made of a total of 15 patients with a mean age of 27.35 ± 9.93 years (range, 17–46 years). The means of arriving at the ED was by ambulance in 10 cases (66.7%) and by other means in five cases (33.3%). The characteristics of the patients are shown in Table 1 and Table 2. Glasgow Coma Score (GCS) was determined as <15 in eight cases (53.3%) and 15 in seven cases (46.7%). Mean systolic blood pressure was 111.60 ± 9.85, mean diastolic pressure 71.46 ± 7.98, pulse 90.60 ± 11.45, and SATO2 95.86 ± 3.75. Laboratory values were determined as glucose, 105.00 ± 22.92, BUN 14.60 ± 5.51, creatinine 0.94 ± 0.16, WBC 8.96 ± 3.02, and pH 11.70 ± 9.92. One patient (6.7%) was admitted to the Intensive Care Unit, and the others were discharged from the ED. Morbidity occurred in one patient (6.7%). The time it took to regain consciousness was 11.70 ± 9.92 h, and the time it took to be discharged from the ED was 20.13 ± 17.03 h.

The year that the SC derivatives were first seen in Turkey is shown in Table 3.

The symptoms and findings of the patients at the time of presentation were determined as anxiety in eight patients (53.3%), palpitations in three (20%), chest pain in four (26.7%), convulsions in eight (28.6%), vomiting in seven (46.7%), loss of consciousness in five (33.3%), mental impairment in five (33.3%), speech impairment in three (20%), hallucinations in four (26.7%), and nystagmus in three (20%). The symptoms of the patients at the time of presentation are shown in Table 4.

## 4. Discussion

The American Association of Poison Control Centres reported the number of cases of SC exposure as 53 in 2009 and 13,000 in 2011 [14]. In a study by Wood et al., cases of SC exposure were reported as 14 in 2009, 2821 in 2010, 6255 in 2011, and an even higher number in 2012 [15]. SC use was first seen in Turkey in 2010, and since then presentations at EDs because of SC use have increased.

In its pure form, SC are substances obtained as a solid or oil. Generally, after breakdown with acetone, they have a plant-like appearance, with various plant parts absorbed. As there are statements on the packaging such as “for aromatherapy use only”, it is perceived as a harmless substance. SC names are usually encoded with letters. For example, they may be labelled according to the name of the person who synthesized them (JWH: John W. Huffman), as well as by music group names (XLR-11), drug company names (A: Abbott Laboratories), chemical names (XLR-11), or website names (RCS) [4,7,14,16]. In studies conducted in Turkey, it was first JWH-018 and then a mixture labelled as CP47 and HU210 that were determined [9].

### 4.1. The Addiction Potential of SC 

The most important problematic feature of SC is their continuously changing new components [12]. SC are four times more effective than cannabis and have a very high risk of intoxication and addiction [12].

### 4.2. The Forms of Use and the Effects of SC 

After intake of SC to the respiratory tract, they are immediately absorbed by the lungs. Following this, they then spread to the brain and other organs in a few minutes, and their effects usually start within minutes [12]. When SC is taken orally, it can be seen that the cause of delayed effects is related to the initial time to metabolism and digestive activity [12]. The SC effect can last for hours. Although SC intoxication is known as a none life-threatening poisoning, death reports have been reported in the literature in recent years [4].

### 4.3. The Clinical Characteristics and Treatment of SC Intoxication 

The majority of cases of acute SC intoxication presenting at EDs are young adult males. To date, the side effects of SC have been reported to cause several complications such as arrhythmia, tachycardia, hypertension, anxiety, agitation, hallucinations, and seizures [17,18]. Moreover, since 2015 an increase has been seen in cases followed-up in ICU and deaths [17,19].

The cases presented in this review reflect similar demographic and clinical characteristics to cases previously documented in the literature. The majority of patients were male and aged below 30 years. The symptoms on presentation were primarily agitation and changes in consciousness. Although there were patients who remained in the ED for a relatively short time, there were also patients who were followed-up for a long period of time. Due to respiratory problems, one patient was died during follow-up. The SC derivatives determined in the dead patient were RCS-04 ortho isomer-M (OH-indole), RCS-04 ortho isomer, and ADB-PINACA-M.

Although acute renal failure was not encountered in the current series, it is recommended in the literature that hydration is applied to patients. In the treatment of acute SC intoxication, intravenous fluids, benzodiazepines, supportive care with oxygen, and anti-emetics, together and symptomatic treatments, are recommended. The majority of the patients in the current series were treated symptomatically with intravenous fluids and benzodiazepines. Intubation was applied to two patients. In five patients, 5 mg intravenous haloperidol was used because of acute psychotic symptoms. The time to correction of changes in consciousness that occurred as a result of the symptoms of intoxication (the time it took to regain full consciousness) was calculated as mean 11.70 ± 9.92 h.

### 4.4. Difficulties in the Management of SC Intoxication

SC have spread rapidly throughout the world and, because of their heterogenous structure, ED physicians experience several difficulties that affect morbidity and mortality. According to a survey conducted among ED physicians, only 20% of the doctors reported that they were prepared for the management of patients intoxicated by SC [20]. The clinical presentation of patients is seen to be heterogenous because of the symptoms related to SC [17,20]. It has been reported in the literature that patients presenting at the ED after SC use often present with aggressive and psychosis-type symptoms [10]. Therefore, patients usually refuse treatment or refuse to give blood and urine samples for laboratory analysis. 

When standard laboratory data are examined, one of the most significant difficulties in diagnosis is that values are within normal limits. In routine drug examinations made for cannabinoids, SC and metabolites may not be determined [9]. Rather than routine tests, SC use was determined prospectively in the current cases with the immunoassay method. Despite the fact that SC use may not be perceived by standard urine drug filters, they may be value in revealing other substances classified together with SC.

In current SC intoxication management, anti-hypertensives are often used for increased blood pressure, and benzodiazepines are used for anxiety and agitation [17,20]. Confirming the substance taken, rather than responding to vital signs or changes in patient behavior, can provide more sensitive and effective treatment [17,20]. It has been stated in the literature that rapid changes in the varying chemical structures of new-generation SC drugs can cause difficulties in treatment because of synthesization in secret laboratories without regulation and control of the production of these components.

### 4.5. The Spread of SC 

To what extent SC are used is not fully known. Due to the relative application of legal restrictions, their ease of acquisition, and the fact that that they cannot be determined with standard substance tests, there is an increasing market demand for these substances.

### 4.6. LC-MS/MS Method and Importance

With the existing LC-MS/MS method, SC have been reliably determined. However, these are advanced tests that are not routinely used in EDs. In recent years, there has been an increase in mortality rates of patients presenting at ED because of SC intoxication [1]. The reliability of the LC-MS/MS screening method used in this study has been confirmed according to the German Toxicology and Forensic Chemistry Association guidelines [21]. The applied LC-MS/MS test has not been fully confirmed. Even if it is not fully confirmed, because there is no commercial reference for commercial production, Clinical samples controlled by LC-MS/MS are promising in assessing the sensitivity of immunoassays in clinical use [22]. In the present study, the new SC types, which were not previously detected in routine tests, were determined by using the LC-MS/MS method (Table 3). Hence, the chameleon-like heterogeneous symptoms and clinical conditions are described in the treatment and follow-up of SC type psychoactive substances. And also SC intoxications are mimic multiple clinical conditions.

## 5. Conclusions

A high rate of false-negative results in SC screening tests can cause errors in diagnosis and treatment. The data of patients screened with the LC-MS/MS screening method in the ED provide an important contribution to treatment planning. As a result of this study, there were three significant findings. Firstly, due to the different clinical forms of presentation at ED associated with SC use, and the range of intoxications that cannot be diagnosed, advanced laboratory tests are required, in addition to routine tests, for the determination of SC. Secondly, those diagnosed as having taken SC were also determined to have used it concurrently with substances that have a high potential for addiction, such as amphetamines and quetiapine. Thirdly, in regard to examples of cases presented in the literature, anti-psychotics, fluid hydration, and anxiolytics can be used as treatment options for those diagnosed with SC use.

## Figures and Tables

**Table 1:** Characteristics of the patients.

Variable		N (%) or Mean ± SD
	Male	14 (93.3)
	Female	1 (6.7)
Age (years)		27.80 ± 12.7
Patients arriving by ambulance		10 (66.7)
Patients arriving with their own transport		5 (33.3)
GCS		
	14<	8 (53.3)
	15	7 (46.7)
Systolic blood pressure (mmHg)		111.60 ± 9.85
Diastolic blood pressure (mmHg)		71.46 ± 7.98
Pulse		90.60 ± 11.45
SATO2 (%)		95.86 ± 3.75
Laboratory values		
	Glucose (mg/dL)	105.00 ± 22.92
	BUN (mg/dL)	14.60 ± 5.51
	Creatinine (mg/dL)	0.94 ± 0.16
	Na (mEq/L)	140.40 ± 1.57
	K (mEq/L)	3.80 ± 0.38
	Cl (mEq/L)	106.10 ± 2.54
	AST (U/L)	24.40 ± 8.14
	ALT (U/L)	23.20 ± 13.98
	Ca (mg/dL)	9.12 ± 0.42
	WBC (103/µL)	8.96 ± 3.02
	pH	7.37 ± 0.21
Time it took to regain consciousness (h)		11.70 ± 9.92
Time it took to be discharged from ED		20.13 ± 17.03
Number of patients intubated and followed- up in Intensive Care		1 (6.7)
Morbidity		1 (6.7)

**Table 2:** Patients’ Characteristics, Treatments and Outcomes.

Case	A	S	BP	P /min	GKS	H	HA	CT (h)	Treatment	SC Type	SAU	Outcome
**1**	24	M	110/60	112	14	+	-	8	IV fluids	5F-AMB, Dihydromephedrone, dihydronormephedrone (mephedrone metabolites)	-	discharged
**2**	20	M	89/59	84	14	+	-	23	IV fluids	5F-AMB, Dihydromephedrone, dihydronormephedrone (mephedrone metabolites)	-	discharged
**3**	63	M	110/80	90	15	+	+	5	IV fluids; haloperidol 5 mg IV”	5F-AMB, 4-APB	-	discharged
**4**	33	M	130/80	74	15	-	-	2	Midazolam 4 mg IV, IV fluids	PX-1, MDMB-CHMICA, ADB-FUBINACA metabolite (artefact deamino-), AB-FUBINACA-M (artefact deamido-)	-	discharged
**5**	33	M	109/77	95	15	-	-	6	IV fluids; haloperidol 5 mg IV”	Dihydronormephedrone (mephedrone metabolite)	-	discharged
**6**	27,	F	110/70	86	15	+	-	7	IV fluids	Dihydronormephedrone (mephedrone metabolite)	-	discharged
**7**	33	M	100/70	86	14	+	-	22	IV fluids	Dihydronormephedrone (mephedrone metabolite)	-	discharged
**8**	21	M	110/70	76	12	+	-	38	haloperidol 5 mg IV; midazolam 4 mg IV; IV fluids; rocuronium 50 mg IV; fentanyl 50 mg IV; fentanyl drip, 50 mg/h; midazolam; 2 mg IV; propofol drip, 5 mg/kg/min	RCS-04 ortho isomer-M (OH-indole), RCS-04 ortho isomer; ADB-PINACA-M (artefact deamino) dihydromephedrone (mephedrone metabolite)	-	Intensive Care Unit, death
**9**	42	M	126/86	105	14	+	-	21	haloperidol 5 mg IV, IV fluids	RCS-04 ortho isomer-M, RCS-04 ortho isomer		discharged
**10**	20	M	120/70	84	13	+	-	8	IV fluids Midazolam 4 mg IV,	5-F-AMB, mephedrone metabolite	+	discharged
**11**	20	M	120/70	86	14	-	-	6	IV fluids	Dihydronormephedrone, Ethylphenidate	+	discharged
**12**	19	M	110/70	104	14	-	-	6	IV fluids	PX-1	+	discharged
**13**	17	M	110/70	85	15	-	-	9	haloperidol 5 mg IV, IV fluids	Ketiapin, mephedrone metabolite	-	discharged
**14**	19	M	110/70	107	15	-	-	11	IV fluids	ADB-FUBINACA	-	discharged
**15**	19	M	110/70	85	15	-	-	5	IV fluids	Ketiapin, mephedrone metabolite	+	discharged

**A** (age, years), **S** (sex; M: male, **F**: female), **BP** (initial systolic and diastolic blood pressure, mm Hg), **P** (initial pulse, beats/min), **H** (history of illicit drug use +, yes, -, no), **H** (history of alcohol use), **CT** (time of consciousness h), **SC** (synthetic cannabinoids), and **SAU** (simultaneous amphetamine use).

**Table 3:** Synthetic cannabinoids (SC) derivatives and metabolites determined using Orbitrap Technology with HR LC-MS/MS.

Synthetic Cannabinoid Derivatives	First Determined in Turkey
RCS-8 (SR-18, BTM-8)	2013
ADB-FUBINACA	2014
Dihydromephedrone	new
PX-1 (PX-1; 5F-APP-PICA)	2015
MDMB-CHMICA; 5F-MDMB-PINACA	2016
ADB-FUBINACA	2014
AB-FUBINACA-M	2013
4-APB	new
5F-AMB	2014
Mexedrone	new

**Table 4:** Symptoms of the patients on presentation.

Symptom	N (%)
Anxiety/Agitation	8 (53.3)
Palpitations	3 (20)
Chest pain	4 (26.7)
Convulsions	4 (26.7)
Vomiting	7 (46.7)
Loss of consciousness	5 (33.3)
Mental impairment	5 (33.3)
Speech impairment	3 (20)
Hallucinations	4 (26.7)
Nystagmus	3 (20)
